# Optimizing the Bluetooth Low Energy Service Discovery Process

**DOI:** 10.3390/s21113812

**Published:** 2021-05-31

**Authors:** Kristof T’Jonck, Bozheng Pang, Hans Hallez, Jeroen Boydens

**Affiliations:** M-Group, imec-DistriNet, Department of Computer Science, KU Leuven Bruges Campus, 8200 Bruges, Belgium; bozheng.pang@kuleuven.be (B.P.); hans.hallez@kuleuven.be (H.H.); jeroen.boydens@kuleuven.be (J.B.)

**Keywords:** bluetooth low energy, service discovery, optimization

## Abstract

Bluetooth Low Energy (BLE), a short-range and low-power communication protocol, has gained a lot of popularity in recent years. A part of BLE is the Generic Attribute Profile (GATT) which defines the data communication between two devices. During the initial connection between two BLE devices a discovery of services, characteristics and descriptors is required for the GATT to operate. During this discovery phase, the device is unusable as it builds the foundation for further data transactions. When unoptimized, this discovery step can take up to a few seconds, leading to frustrations for the end user or delays in some applications. In this paper, we aim to find guidelines on how to optimize this discovery process. A simulation framework was developed, able to simulate and analyze the packet exchange of the service discovery, while taking link layer parameters into account. The results show that minimizing the connection interval and maximizing the data length leads to the lowest discovery times. Practical experiments in real environment, however, show that the theoretically calculated times are not reachable due to processing overhead and retransmissions. Theoretical results also show that the current BLE discovery process, even after optimizations, has a lot of overhead. To fix the problems with the current protocol, this paper proposes a new Rapid Service Discovery Protocol, which enables a fast and efficient service discovery.

## 1. Introduction

During recent years, IoT technologies and sensor networks have gained a lot of attention. More sensors are getting embedded into wireless devices, thus making them smart devices. These devices and networks are increasingly being used in various kinds of domains such as industry, healthcare, and consumer products. To enable data transfer between sensor devices, a sensor network with a specific wireless communication protocol should be used. Many wireless technologies for short-range communication are currently on the market, such as Bluetooth, Wi-Fi, Ultra-Wideband, or Zigbee. All these technologies have their own specifications, which are mostly trade-off between several factors such as throughput and energy consumption [1]. Another short-range protocol which has gotten a lot of traction in recent years is Bluetooth Low Energy (BLE).

The BLE specification is being actively developed by the Bluetooth Special Interest Group (SIG), leading to updates of the specification and addition of new features. BLE already has widespread support in daily-used devices such as laptops and smartphones, allowing easy integration. Due to its low price, low power and built-in security, BLE is an attractive choice in different kinds of scenarios. Recent research shows the applicability of BLE in different contexts such as industry [2], healthcare [3,4], home automation [5].

As its popularity grew, research has been performed to analyze, improve and verify the efficiency and the performance of the Bluetooth Low Energy stack. Gomez et al. [6] presented an overview of the BLE specification [6]. They analyzed the performance of the BLE stack, in terms of energy consumption, latency, pico-net size, and throughput. A theoretical and experimental study was made, investigating critical parameters which influence its performance, such as the connection interval and connection slave latency. Tosi et al. [7] have made a systematic review of the research already done recently about the performance of BLE [7].

With BLE claiming to be a low energy protocol, some studies have focused on its energy aspect. While Kindt et al. [8] have modelled the energy consumption of the Bluetooth stack, Siekkinen et al. [9] performed a comparison based on energy efficiency between BLE and other low energy protocols, such as Zigbee [8,9].

As its popularity grows and more and more systems are deployed using BLE, its reliability is being researched. Pang et al. [10] study the impact of the increasing number of devices and the impact of different radio frequency environments on BLE reliability [10,11]. Similarly, the work of Spörk et al. [12] proposes improvements for the BLE stack to deal with the interference and thus improving the reliability of the spectrum [12].

Finally, other research has focused on improving the speed of the BLE stack. In the study by Liu et al. [13], different ways to increase the discovery speed of devices have been researched [13,14]. Through simulations, Mikhaylov [15] proposes an optimization to accelerate connection establishment [15,16].

As shown in previous paragraphs, most state-of-the-art research focuses on analyzing and improving lower layers such as the physical and link layer of the BLE communication protocol, hence the performance of higher layers of the BLE stack are not considered yet. Specifically, these parts could use improvements. Little to no research has been done on the service and characteristic discovery protocol of BLE, which is an important step during the initial connection of a BLE device.

To achieve a low-power data transfer, Bluetooth Low Energy uses a protocol named the Generic Attribute Profile (GATT) which makes use of the underlying Attribute Protocol (ATT). This ATT protocol has a hierarchical structure of services. These services, in turn, contain characteristics where data are stored. Data transfer is enabled by a central/peripheral type of connection, where the central requests data from or sends data to a peripheral device via GATT.

A service discovery process is required to enable the data transfer between both devices. This service discovery will discover all the capabilities of the peripheral device by requesting its attributes. The service discovery process thus contains a series of requests and responses from central and peripheral.

As many devices in the field become more complex, the number of services and characteristics also increases. This, in turn, leads to an increase in the time needed for a full service discovery. During the discovery process, the device is unusable, as it is building the foundations for future data transactions. In dynamic environments where devices often reconnect or environments which require service rediscovery at runtime, this could introduce unwanted delays. This delay depends on the used BLE parameters and can be up to tens of seconds in the worst case. Reducing the discovery time to the minimum is a must in such circumstances. Additionally, in other applications with human interaction, the user expects to be able to use the BLE device instantaneously. Research has shown that with a reaction speed of under 0.1 s a user perceives it as instantaneous [17,18]. Optimizing the service discovery will lead to small waiting times on the initial connection, thus increasing the user experience considerably.

This paper aims to optimize this service discovery by finding the optimal parameters in the protocol. These are parameters from the Bluetooth stack which can be changed during runtime and define the speed and size of the messages in the packet exchange. First, a framework is made which can theoretically calculate the time needed for a full service discovery. Secondly, a physical implementation was made. Time measurements of the practical setup are then compared with the theoretical results. Finally, we propose a Rapid Service Discovery Protocol enabling faster discovery and rediscovery.

The rest of the paper is structured as follows. Section 2 provides a more in-depth explanation of the service discovery process as defined in the current Bluetooth Specification. Section 3 gives a theoretical analysis on how each of the parameters influence the speed of the service discovery. In Section 4 we compare these results with a practical implementation. In Section 5 we then propose a novel service discovery protocol, speeding up the current implementation. The importance, relevance, and limitations of this research are shown in Section 6, the discussion section. Finally, the conclusions are drawn in the conclusions section, Section 7.

## 2. Background

The discovery protocol of BLE is a part of the Bluetooth Low Energy specification which works on top of other different layers. This section gives a background of the relevant information in order to fully understand the Bluetooth Low Energy discovery protocol. The principle of BLE connections, packet exchange, and packet transmission are explained which enable the discovery protocol to work. With this information a detailed description is given of this protocol together with the parameters which influence its performance.

### 2.1. Bluetooth Low Energy

The BLE wireless technology has been added to the Bluetooth specification since Bluetooth 4.0 working on the same 2.4 GHz ISM (Industrial, Scientific, and Medical) band as other well-known wireless protocols such as Wi-Fi, Zigbee and Bluetooth. These protocols have the advantage that they are well adopted, and devices increasingly support these technologies [19]. Most recent devices, like laptops, smartphones, … already have a Bluetooth chip supporting Bluetooth Low Energy enabling interoperability between many existing low energy devices. Not only are the interoperability and active development advantages of the technology, but also the low cost and its low energy consumption are factors making it a popular option [20].

The BLE stack itself is built upon many layers which enable an efficient and secure data transfer. To fully understand the discovery protocol, more knowledge is needed of some of these layers which define how BLE connections are made and how data transfer is achieved.

### 2.2. BLE Connection

Communication between two BLE devices can happen in two distinct ways. The first way is via the broadcasting mode which is connectionless. The second way to communicate is via a connection. As service discovery only happens in a connected mode, we will not go into further detail of the broadcasting mode.

A connection is a link between two BLE devices which periodically exchange packets between each other. Within a connection there are two roles defined in the Generic Access Protocol (GAP), the layer responsible for handling these connections. These two roles are:

- A central device will discover peripheral devices by listening for advertisements from these devices. The central can then initiate a connection which allows communication between both devices. The central device can also be referred to as the master or client.
- A peripheral device broadcasts advertisement packets to make itself discoverable. A central master device can respond to these advertisements to initiate a connection with this device. The peripheral device can also be referred to as the slave or server.

When the central device responds to the advertisements to set up a connection, the connection request can be accepted by the peripheral and connection will be established. After the establishment of a connection, the master and slave will start to communicate and start to exchange connection parameters. These parameters define different link characteristics such as timing intervals, physical layer properties and data length properties. After these connection steps, generally, a discovery of services and characteristics occurs to enable further data transfer (see Section 2.6).

### 2.3. Attribute Protocol

To achieve a low-power data transfer between two devices, Bluetooth Low Energy uses a protocol named the Attribute Profile (ATT). The attribute protocol is responsible for transmission and storage of data between a peripheral and central device. The data are stored into a structured attribute table where each attribute contains four fields:

- Attribute Handle: a unique handle number specific to that attribute in the attribute table. The value of this handle lies within the range of 0x0001 and 0xFFFF. Gaps are allowed between succeeding handle numbers in an attribute table, although they must be in an increasing order.
- Attribute Type: the type of the attribute which is defined by a specific Universally Unique Identifier (UUID).
- Attribute Permission: the permission which allows the access to read or write to the attribute.
- Attribute Value: the value of the attribute. This can be various kinds of data.

An example of a part of such an attribute table is shown in Table 1.

Next to the data storage in an attribute table, the ATT protocol is also responsible for data transfer of the attributes via methods to read and write the data. This is possible via commands, requests, responses, notification, indications and confirmation messages defined by the ATT.

The Generic Attribute Profile (GATT) is a layer which works on top of the ATT and uses it to exchange data between devices. Similar to the GAP, the GATT also distinguishes two roles: the server and the client. These respectively match the peripheral and central roles from the GAP.

The GATT gives a higher abstraction of the ATT by using the attributes to provide a hierarchical representation of services, characteristics, and descriptors. Predefined use-cases such as heart rate, blood pressure and battery level, are standardized in specific profiles. These profiles contain the necessary attribute structure of services and characteristics and descriptors for that specific application. These profiles enable interaction of devices from different vendors and makes them interoperable with each other.

GATT also enables data transfer within a connection between a central and peripheral device. The central device is the master and requests data from, or sends data to, a peripheral device. A detailed explanation of this protocol is given in Section 2.4.

As an example, a peripheral heart rate device is taken which simulates a heart rate device on the body of a person. A central device or GATT client can communicate with this device in order to retrieve data from the peripheral device.

Each of these services has its own characteristics and descriptors defined. Table 1 shows a small part of such an attribute table where a heart rate service is defined according to the heart rate profile. This attribute table shows entries for the heart rate service with a Heart Rate Measurement and a Body Sensor Location characteristic. The Heart Rate Measurement characteristic, in turn, has a mandatory descriptor, the Client Characteristic Configuration Descriptor (CCCD), which is used to turn on the notifications.

All of these previously mentioned services are basic standardized services which are defined within the Bluetooth Low Energy specification [21]. This means that these services are defined in advance with a predefined Universally Unique Identifier (UUID). All UUIDs in Bluetooth Low Energy have a length of 128 bits. The standardized services, characteristics, and descriptors are defined in the Bluetooth Low Energy stack as a shortened 16-bit values. As the UUIDs must be a 128 bit all the predefined attribute UUIDs will be integrated in the form of a base UUID XXXXXXXX-0000-1000-8000-00805f9b34fb A Heart Rate service for example has the UUID 0x180D which leads to a UUID of: 0000180d-0000-1000-8000-00805f9b34fb.

An advantage of using these predefined UUIDs, is that these 16 bits can be used during the service discovery instead of the complete 128 bits. Not all services, characteristics and descriptors that people might need can be included in the specification. For services which are not defined by the Bluetooth Low Energy stack, custom UUIDs can be created. These custom defined UUIDs can use all the remaining 128-bits UUIDs apart from the previously mentioned base UUID. The UUIDs during packet transfer are solely used during the advertising and service discovery process. Using the 16-bit standardized services, characteristics and descriptors leads to less packet traffic which in turn leads to less time spent on data transfer and lower power consumption during the discovery phase. If using a custom defined UUID for a service/characteristic all the 128 bits must be sent during these steps leading to more overhead.

### 2.4. Packet Transmission

**Figure 1:**
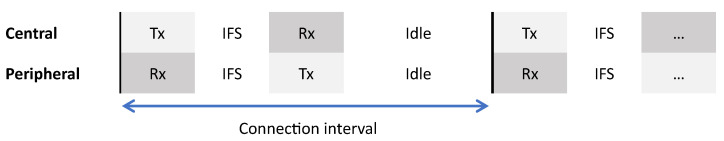
Packet exchange.

The packet exchange within a Bluetooth Low Energy connection is shown in Figure 1. The connection between a peripheral and central device consists out of consecutively alternating packets between each other. It is always the central device that starts the transmission. Upon the initiation of data transfer, the clocks of the central and peripheral devices get synchronized. This synchronization point is called the anchor point. The time between two consecutive anchor points is the connection interval. During this time central and peripheral always alternate between receiving and sending until both central and peripheral have no more packages to send. The More Data (MD) bit in the link layer header indicates whether more data has to be transmitted. If both central and peripheral indicate in their last package that they have no more data, both devices will go to sleep until the next connection interval. The duration within one connection interval where actual data transfer is happening is called the connection event. Note that it is always the packet of the peripheral that will close the connection event.

Between each alternating packet there will be an inter-frame space (IFS). This IFS is a delay which prevents collisions from happening and has a fixed time of 150 μs. The length format of the packet itself depends on the choice of physical mode and data length.

### 2.5. Packet Format and PHY Modes

**Figure 2:**
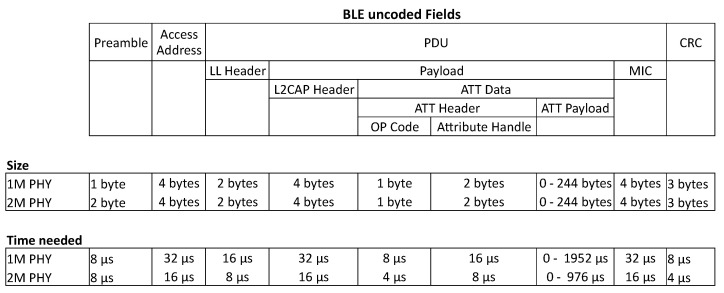
Uncoded packet structure.

Bluetooth low energy has four physical layer transmission modes with each their benefits. In Bluetooth versions lower than Bluetooth 5, the physical layer was fixed and used the 1M transmission mode, which is able to send data over at a speed of 1Msymbol per second. As this transmission is uncoded it will send with a speed of 1 Mbit per second. Bluetooth 5 introduced a new physical mode with a double symbol rate, increasing the transmission speed to 2 Mbit per second. This mode is called the PHY 2M mode [22]. Both of these modes have a similar packet structure and are uncoded. This structure is shown in Figure 2.

In Bluetooth 4.0 and 4.1, the data length of the payload is fixed to 27 bytes. Starting from Bluetooth version 4.2 there is a new feature called Data Length Extension (DLE) which allows the payload to be extended up until 247 bytes. The Maximum Transmission Unit (MTU) parameter from the Bluetooth stack then decides the actual data size of the Attribute Data. The MTU size is standard 23 bytes which means a payload length of 27 bytes (L2CAP headers 4 bytes + ATT Data 23 bytes). There is no limit defined in the Bluetooth stack of this MTU size, although most Bluetooth stacks limit this. If an MTU of 247 is used it will use the largest packet size supported by the data length extension. If the data length is set lower than the MTU size, the L2CAP layer will fragment the data in different packets and will reform it when the packets are received.

Since Bluetooth specification 5.0, a new physical mode has been introduced, named the BLE-Coded PHY [22]. This mode makes it possible to achieve longer ranges due its more robust communication protocol. Additionally, another advantage of this protocol is the robustness it achieves in noisy environments or environments with obstacles.

**Figure 3:**
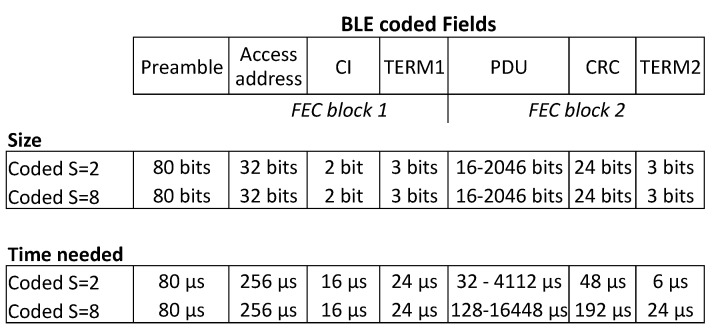
Coded packet structure.

The Link Layer format of the LE Coded PHY will be different than the traditional BLE uncoded structure to provide more resilience to noise. A picture of the packet structure can be found in Figure 3.

Two encoding schemes can be used with BLE Coded: S=2 and S=8. FEC block 1 will always be encoded using the S=2 encoding scheme. The second block depends on the chosen encoding scheme. When S=8 coded is used each data bit in FEC block 2 will be represented by 8 symbols, whereas when S=2 coded is used each data bit will be represented by two symbols. The symbol rate will be 1 Msps, leading to data rates of around 125 kbps and 500 kbps, when using LE Coded with S=8 or S=2 respectively.

### 2.6. Discovery Protocol

To make use of GATT data transactions, a central device must discover all the services, characteristics, and descriptors of a peripheral. To do this, the central will send a series of requests to be able to recreate the attribute table (see Section 2.3) of the peripheral device. Future data transactions will be based on the information received during this discovery.

**Figure 4:**
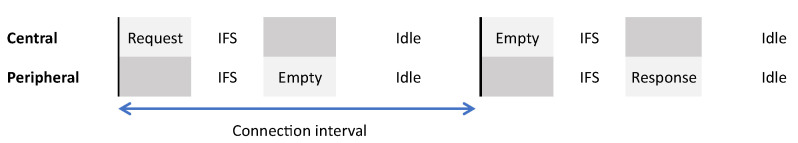
Typical discovery request and response.

Figure 4 shows how a typical request of the GATT discovery protocol in Bluetooth Low Energy looks like. It starts with a request from the central device asking for info from the peripheral. As the BLE specification dictates both central and peripheral need to send a packet without the more data bit set to end the communication in that connection interval. The peripheral will send an empty packet in that same connection interval to end the connection event. In the next connection interval, the central starts with an empty packet to initiate a new connection interval and connection event. The peripheral will send the response of the request from the previous connection interval while ending that connection event if it has no more data to send.

**Figure 5:**
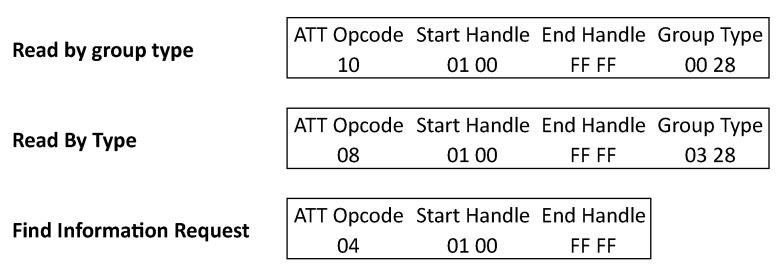
Discovery requests.

**Figure 6:**
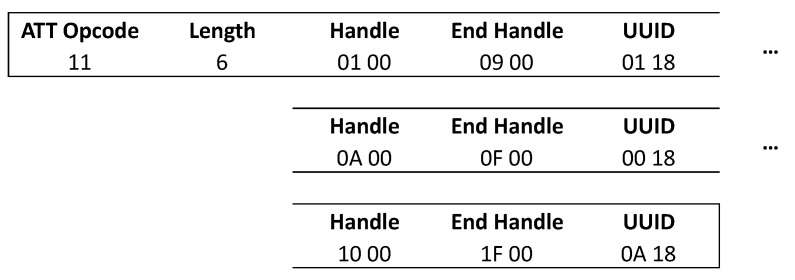
Service discovery response.

Figure 5 shows an overview of the different possible requests needed to perform a full-service discovery. A read by group type request is done to search for the Primary GATT services in the peripheral. These Primary GATT services represent the primary functionality provided by the peripheral device. There also exist secondary services to be included in the primary services. We will not discuss these secondary services as they are rarely used in practice. This is initiated by the central, in this example it is requesting the services with a handle from 0x0001 till 0xFFFF, which is the full range of all handles. The peripheral will then respond with the services within that range of handles. A response packet holds an opcode of the service response. It also contains the length of each attribute defined in that packet. Since Bluetooth Low Energy specification 4.2, it is possible to increase the length of the data payload in the link layer from 27 up to 251 bytes by enabling the LE Data Packet Length Extension. The number of services fitting in one packet is also dependent on the ATT_MTU (attribute maximum transmission unit). When the MTU is higher than the allowed bytes of the data length extension, fragmenting will be used to send bigger packages in the link layer [23]. If all the services do not fit into a single packet, the master must request the other services starting from the end handle of the last received service. This until an “attribute not known” response is sent by the peripheral, which indicates that the service discovery is finished. An example of a response to the “read by group type” request is shown in Figure 6. Here each service attribute consists of 6 bytes. This is the size needed to send the details of the service with a standardized predefined 16-bit UUID. The first 2 bytes are used to define the starting handle of the service, the next 2 bytes define the end handle, and the last 2 bytes of the six define the 16-bit UUID. All the characteristics which belong to that service will be within that range in the attribute table. In a standard size packet of 23 bytes, three standardized services will fit. If a custom UUID is used 20 bytes are needed, two for the start handle, two for the end handle and 16 for the UUID, meaning only one service can be sent per packet when using the standard data length of 27.

**Figure 7:**
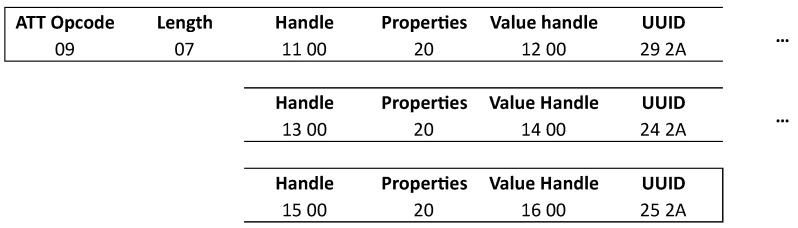
Characteristic discovery response.

Once all the services are discovered, a characteristic discovery can happen. To do this request a similar approach is used as the service discovery. It uses the “read by type” request, again with a start handle and an end handle to request the characteristics. When discovering the complete attribute tree, the choice can be made to use the previously discovered service handles to discover service per service. An other option is to discover everything at once by again putting a start handle of 0x0001 and end handle of 0xFFFF in the request. The peripheral will respond with the handle, properties, value handle and characteristic UUID of the characteristics. Figure 7 shows the structure of this package. When custom characteristics are used, the length will be 21 bytes.

**Figure 8:**
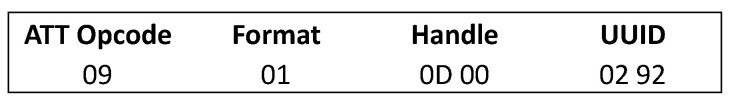
Descriptor discovery response.

Finally, the descriptors can be discovered again with a start and end handle via a “find information request”. Figure 8 shows an example format of the descriptor answer. The format field will define whether it is a 16-bit descriptor or a custom descriptor. The descriptors can often be evaluated without any discovery. This due to fact that the specification dictates that the descriptor must be defined when using indications or notifications.

## 3. Theoretical Analysis

With the knowledge from the Bluetooth specification, a theoretical framework is made to simulate, calculate, and optimize the time of the discovery process. In a discovery process, three things are queried: the services, the characteristics in each service and the descriptors in each characteristic. In essence, the time to do a complete discovery is the sum of the time of the primary service discovery, characteristic discovery, and descriptor discovery.

As mentioned before in Section 2.6, the discovery happens with an alternation of requests and responses between central and peripheral.

The BLE specification provides a way to discover all services via a request which queries all services at once. According to the specification, a characteristic discovery should happen for each service separately. This can be avoided by also requesting all characteristics at once, thus reducing time overhead. For the service and characteristic discovery, if not all services/characteristics fit in one packet the central device will again request the next services/characteristics. Once the peripheral sends the “Attribute not found” packet, all necessary attributes are discovered.

The descriptor discovery cannot be done all at once and has to be queried per handle range which was not discovered yet in the protocol. Due to the limitations in the current specification, it is not possible to do a full descriptor discovery at once. Many applications only use the CCCD descriptor which is mandatory for characteristic notify and indicate operations. By checking the indicate-and-notify properties of previously-discovered characteristics, the descriptor discovery can often be skipped. During the simulation we include a discovery for all descriptors.

**Figure 9:**
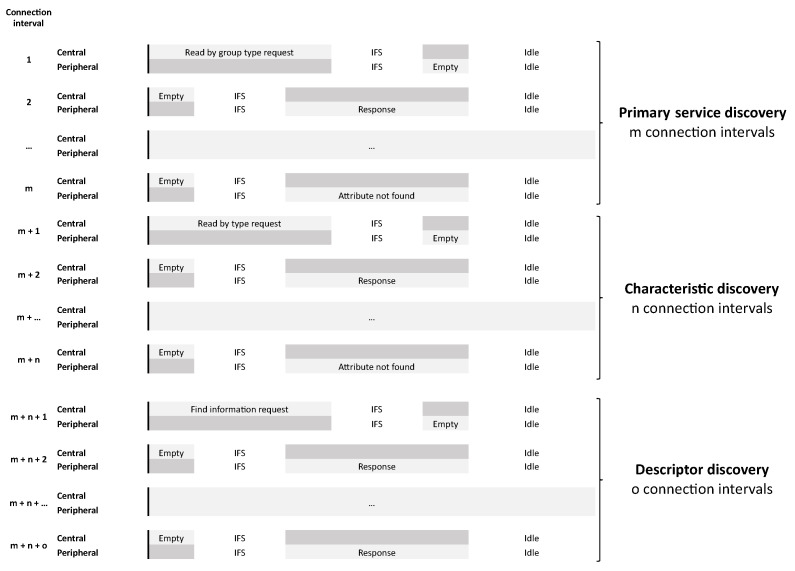
Full service and characteristic discovery sequence.

With all the received information the central can reconstruct the whole attribute table from the peripheral device. In Figure 9 an overview of a complete discovery sequence is shown.

There are three parameters that can influence the timing of the discovery protocol, which can be found in the BLE specification. These parameters are: the connection interval, the MTU/Data Length, and the Transmission speed/PHY mode.

The following section shows how a simulation framework was made able to test the influence of these parameters during the service discovery. The sections thereafter give a more detailed explanation of how each parameter affects this discovery.

### 3.1. Simulation Framework and Simulation Setup

To make accurate calculations of the service discovery, a simulation framework was made using Python 3.8.

Using the framework, peripheral devices can be generated with different services, characteristics and descriptors. The framework is able to generate the necessary packets according to the specification to do the full discovery of all the services, characteristics and descriptors.

As mentioned before, the time-of-discovery protocol is dependant on the parameters of the Bluetooth Low Energy link layer. There are three parameters that can influence the timing of the discovery protocol, which can be found in the BLE specification. These parameters are: the connection interval, data length/MTU and transmission speed. All of these parameters are considered during the simulation of the service discovery process. A list is made of packets and connection intervals taking into account the link-layer parameters. The simulation framework additionally keeps track of the time distribution of the service discovery. By doing this the efficiency of time users can be calculated.

To check the influence of each parameter, a theoretical calculation is made by gradually increasing each parameter within their possible range. The time needed to do a full discovery is heavily dependant on the number of services, characteristics, and descriptors a peripheral contains. For that reason a simulation is made for two distinct devices. A device containing five services, 12 characteristics and three descriptors (referred to as Device 1) and a significantly larger device with 50 services, 120 characteristics, and 30 descriptors (referred to as Device 2). By making an analysis for both devices, the impact of the number of services, characteristics and descriptors can be shown. The following sections explain the results of each of the connection parameters and how they affect the discovery speed for both devices.

### 3.2. Connection Interval

The connection interval, as explained in Section 2, determines the time between two connection events. In the current BLE specification, it requires two connection intervals for each service discovery packet sent.

**Figure 10:**
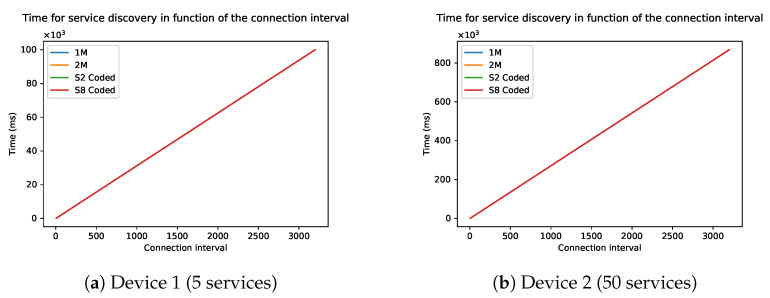
Time of the discovery process as a function of the connection interval of two devices: (**a**) Device 1 and (**b**) Device 2.

Figure 10 shows the influence of the connection interval on the total discovery time starting from the minimum value 6 ranging until the maximum value of 3200. Note that the time on the Y-axis is expressed on a scale of thousands. The curve of the graph is linear because the number of packets stays the same. Each connection interval will only be used to send one packet, but the idle time in each connection interval will increase. With the lowest interval of 6 (7.5 ms) it will take a few hundred microseconds for the discovery of device 2, up to more than 400 s when the interval is at its maximum of 3200 (4 s). Device 1 will also take hundred milliseconds in the best case and a few seconds in the worse case. The data length of each packet is kept at 27 during this simulation, which is the standard packet length. The figure also shows that the physical mode (PHY) has little to no influence on the time, as all curves are on top of each other.

### 3.3. Maximum Transmission Unit and Data Length

As explained before, the data length and maximum transmission unit (MTU) will determine the maximum payload of a packet. A bigger payload means more services and characteristics fit in a single packet. This also means that fewer packets must be sent to do a full discovery.

**Figure 11:**
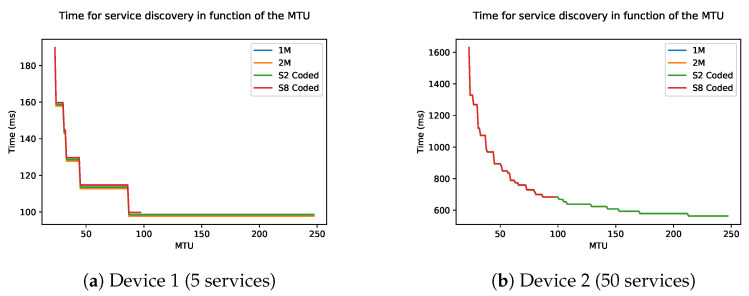
Time of the discovery process as a function of the MTU of two devices: (**a**) Device 1 and (**b**) Device 2.

Figure 11 shows the time in function of the data length, which ranges from 27 to 251. The connection interval is kept constant at 6, as previous simulation has shown that a lower connection interval causes the fastest discovery. The figures show stair structure. One step of the stair means that more services can be grouped together in one packet, leading to fewer packets and thus less time. In general, this means that the larger the maximum data length, the faster the discovery. The physical mode of the BLE connection has a small influence on the discovery speed. This difference is due to the length of the last packet which is dependant on the physical mode. This difference is more noticeable in devices with fewer attributes, which in turn have fewer packets to be sent.

### 3.4. Time Analysis

**Figure 12:**
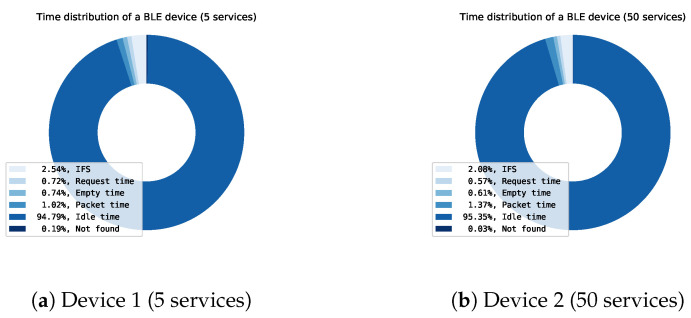
Time distribution of the discovery process of two devices: (**a**) Device 1 and (**b**) Device 2.

The discovery protocol can be optimized by using the optimal settings (Connection interval: 6; Data length: 251; Physical mode: 2M) gathered from previous results. When these settings for both Device 1 and Device 2, a time distribution analysis can be made of the BLE communication. Figures of the time distribution of Device 1 and Device 2 can be found in Figure 12. These figures are created by using our simulation framework.

The time analysis shows the time distribution of the discovery protocol. The time can be split into six sorts of data:

- IFS: The inter-frame spacing, which is the time between two subsequent messages
- Request time: The time used by the central for requesting services, characteristics, descriptors of a peripheral device
- Empty time: Time used by central or peripheral devices when sending empty packets.
- Packet time: Time used by the peripheral device to actually send the requested service, characteristic or descriptor data.
- Idle time: Time where both the central and peripheral device are idle.
- Not found: Time used by an “Attribute not found” packet. The peripheral sends this packet to indicate if he has no services/characteristics within the requested handle range. The central then knows that all services/characteristics have been discovered.

The useful data is the actual packet data containing services, characteristics and descriptors. The rest of the time (IFS, Request time, Empty time, Idle time and Not found) is pure overhead. In a device with a smaller number of services (Device 1), the actual useful time (Packet time) is only around 1% of the total time. When looking at Device 2, the useful packet time increases to 1.37% which is still very small compared with the total time. When analyzing these results further, it can be noticed that even after optimization, the largest chunk of time (95%) is idle time. This indicates that there is a need for a better discovery protocol, able to better fill the idle time.

## 4. Experimental Analysis

To validate the findings of the theoretical analysis a practical setup is made. An implementation of different settings and devices is created exactly like examined in our simulation framework. By doing this, a direct comparison can be made between theoretical and practical results.

### 4.1. Testing Methodology

**Figure 13:**
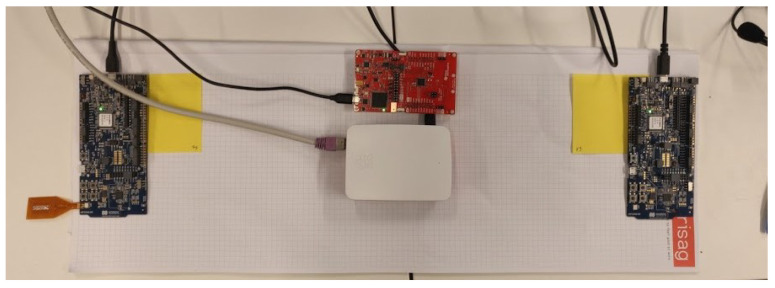
Practical measurement setup: on the left side an nRF52 central device, on the right side an nRF52 peripheral device, and in the middle a TI CC2652RB Sniffer.

To perform the tests between devices, a Bluetooth Low Energy central and peripheral are made. To capture all the packets between the central and peripheral a BLE sniffer [24] is used. The sniffer used is the nRF Sniffer for Bluetooth LE together with an nRF52840 development kit [25] of Nordic Semiconductors. The nRF52 sniffer does not provide support for the Coded S8 and S2 physical modes, an additional sniffer is used. The additional sniffer is a TI CC2652RB Launchpad Board [26] which uses the “Sniffle” [27] python library. In Figure 13 the practical setup is shown with the TI CC2652RB sniffer in the middle between a central and peripheral device.

During the experiments, a peripheral and central device establish a connection with different BLE parameters. The sniffer then captures all the packets transferred between both devices and then saved in a Pcap file which can be opened by Wireshark. The captured packets contain detailed information about the complete packet structure of the BLE packets which were transmitted. Using these details we can see how a connection is set up, and how long a discovery takes in practice. The packet information also includes the timestamp of each packet. To calculate the discovery time a script was written in python. This script uses the pyshark [28] library to parse the Pcap files and calculate the time of the discovery process.

### 4.2. Experiment 1: nRF52 Central with an nRF52 Peripheral

In the first experiment, both the central and peripheral are built using an nRF52840 development kit. A real time operating system (RTOS) is used on these devices, called Zephyr RTOS. This open-source operating system is widely adopted by many players in the market such as Intel and Nordic Semiconductors and has an extensive BLE stack.

To get the practical results, implementations of peripheral devices are made with the same services and characteristics as simulated in the theory. A first device with a total of five services, 12 characteristics and three descriptors, is referred to as Device 1. A second device, with 50 services, 120 characteristics and 30 descriptors, is referred to as Device 2. Data are captured in different settings such as MTU, connection interval and physical mode.

In Table 2 an overview can be found of all the practical results. It also contains the theoretically-calculated time and the percentage difference between the practical discovery time and the simulated time for Device 1.

The results show that the fastest time for service discovery is indeed acheived with a maximum MTU and minimal connection interval. The used physical mode has little to no influence on the discovery time. A small difference between theory and total time can still be noticed. To get understand what causes this difference, we took a deeper look into the sniffed packets. There we see that some packets were lost and retransmitted due to interference in the office environment. When using an MTU of 247, the percentage difference is often higher. This difference is due to the higher probability of interference for larger MTUs. Additionally, due to the fact that fewer packets have to be transmitted with a higher MTU, one lost packet has will have a larger impact on the total time. Despite these delays, the discovery time when using a large MTU will still be a lot faster than with lower MTUs. With a connection interval of 6 (7.5 ms), physical mode of 1M and an MTU of 23, there is a percentage difference of only 0.33%. For a connection interval of 60 (75 ms) with a physical mode of 2M and an MTU of 247 there is only a difference of 0.04%. When looking at the sniffed packets during those connections, no retransmissions or other delays were found. These minimal differences are due to the timing errors of the BLE sniffer. These experiments were repeated with Device 2 to see how it reacts in an office environment. Results are shown in Table 3.

The results for Device 2 are similar to those of Device 1. However, the percent differences are more diverse. Since there are many more services, characteristics, and descriptors, more packets are sent. Thus, a single packet retransmission will have a smaller impact on the total time. During these experiments the physical mode also had little to no influence on the results. The largest MTU and lowest connection interval also showed the best results for Device 2.

During the performance of these practical tests, other points of attention also emerged that should be taken into account. The processing overhead still needs to be taken into account. In the event that processing occurs during the discovery process, it should be taken into account that these processes take less time than a connection interval. By doing this, we can guarantee that the delay will only be caused by retransmissions and not by additional processing. In our experiments, we optimized the processing by eliminating all overhead, such as debugging, to make sure it did not affect our discovery.

### 4.3. Experiment 2: Android Peripheral with nRF52 Central

To see the difference between BLE stacks, an implementation of peripheral Device 1 was made using android. A connection was set up with the nRF52 central of Experiment 1. When using the sniffer to receive these packets, a noticeable difference can be spotted in these packets. The peripheral fits three characteristics into a characteristic discovery request as opposed to zephyr RTOS, only putting two into a response. This could be due to the interpretability of the BLE specification. In the BLE specification they state that the Attribute data consists of an attribute header of 3 bytes and an attribute payload of maximum 244 bytes. When using an MTU of 23 the attribute data should be maximum 23 bytes meaning only 20 of attribute payload can be used. The service discovery, however, only uses one byte as attribute opcode and one byte as the length, meaning 21 bytes remain when using an MTU of 23.

To summarize we can say there are two options:

- When interpreting this packet as an attribute protocol packet and strictly following the format. This means that only two characteristics fit into the packet when using 23 as MTU.
- When not strictly interpreting and following the packet format as an attribute protocol packet, three characteristics fit into a packet when using 23 as MTU.

### 4.4. Experiment 3: Android Central with nRF52 Peripheral

After Experiment 2 we also test if the central stack shows any differences in Android. We implement the Android device as central device and an nRF52 peripheral device. During the implementation and optimization process of the central Android device, a lot of limitations arose.

Firstly, service and characteristic discovery are regulated by a high-level software stack that encapsulates the lower layers. When using the Android API we have to stick to this limited specific programming interface. With one single instruction “discoverServices()” it discovers all services, characteristics and descriptors. When looking at the sniffer it first does a service discovery and then does a separate request for each service to discover the characteristics within its range and then discovers the descriptors for the characteristics. This method is not optimal as it cannot group all characteristics together in one packet when using a large MTU.

Secondly, the parameters are enforced by the central android side. The parameters of the peripheral device are rather suggestive as a central can choose to ignore these preferred peripheral settings. In Android, it is not possible to set a specific Bluetooth Low Energy connection interval. Since Android Lollipop or newer, there is an option to change the connection priority [29]. It can be found that changing the connection priority changes the interval to a specific range, by diving deeper into the source code of the Android BLE stack [30]. Three values are possible:

- CONNECTION_PRIORITY_HIGH: 9–12 (11.25–15 ms)
- CONNECTION_PRIORITY_BALANCED: 24–40 (30–50 ms)
- CONNECTION_PRIORITY_LOW_POWER: 80–100 (100–125 ms)

When running some test with the sniffer, we can see the Android stack temporarily changes the connection interval to 6 (minimum time of 7.5 ms) to increase the speed. In theory doing this should drastically increase the speed. Although, in practice, we see that the connection interval parameter takes effect in the middle of the discovery process.

## 5. Rapid Service Discovery Protocol

As the normal service discovery process has disadvantages, such as the waiting time between connection intervals and the overhead of the request packages, we propose a new discovery protocol to be used alongside the other discovery protocol. This Rapid Service Discovery Protocol (RSDP) eliminates the overhead while still ensuring that all services are discovered.

The results of our theoretical analysis (see Section 3.4) show that the useful packet time is too low in comparison to the overhead. To increase the speed of discovery, the overheads (IFS, Request time, Empty time, Idle time and Not found) have to be decreased. The time for most of the packets is fixed such as IFS, request time, empty time and not found time. To lower the total time, the occurrences of these packets need to be decreased.

**Figure 14:**
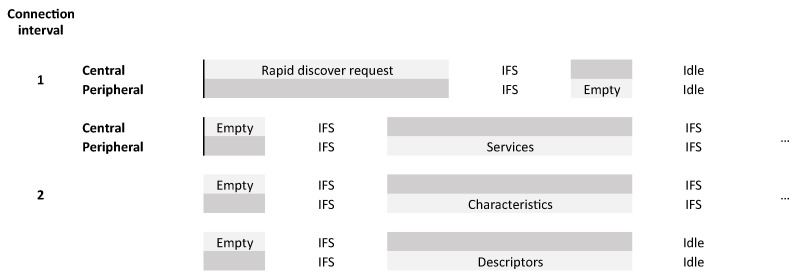
Rapid service discovery protocol.

The protocol is shown in Figure 14. The RSDP only requires a single request. The peripheral in turn will try to respond with its services as it would normally do. Instead of sending only one packet, it could fill up as many packets as it can in that single connection interval. If all services have been sent, an empty attribute with handle 0xFFFF is added as service to show that all services have been sent. The characteristics and descriptors are sent in the same manner within the same connection interval. Using our simulation framework we simulated and calculated the time needed for the RSDP on the same devices as in Section 3.

### 5.1. Physical Mode, Connection Interval and MTU

**Figure 15:**
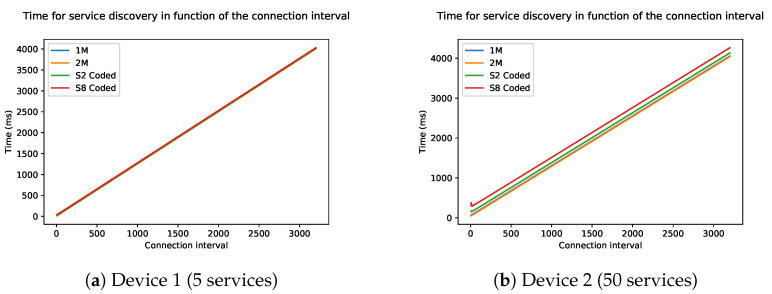
Time of the RSDP as a function of the connection interval of two devices: (**a**) Device 1 and (**b**) Device 2.

Figure 15 shows the time needed to do the service discovery with the RSDP. The physical mode has an influence on the performance. The time needed for an S8 Coded packet will be drastically longer than one sent with a 1M physical mode, which means fewer packets fit in one connection interval.

**Figure 16:**
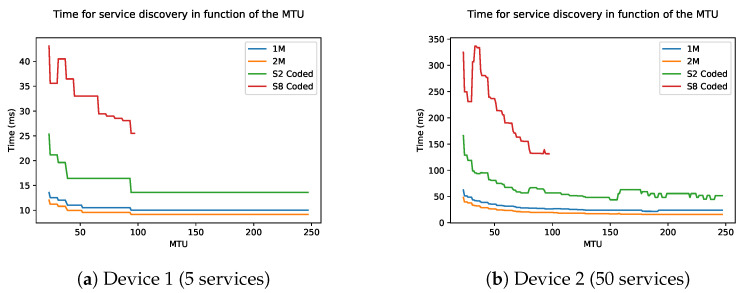
Time of the RSDP as a function of the MTU of two devices: (**a**) Device 1 and (**b**) Device 2.

Figure 16 shows the influence of the maximum data length on the total discovery time. The connection interval is set to a minimum of 6 (7.5 ms). As previously mentioned, the physical mode will have an influence on the time. The total discovery time again decreases when using larger data packets with Device 1. With Device 2 the time sometimes fluctuates. This is due to the fact that when packets become larger, the packet cannot fit into the connection interval anymore. When this happens, it has to wait till the next connection interval to send this packet, causing an extra time overhead.

### 5.2. Limitation of Packets per Connection Event

**Figure 17:**
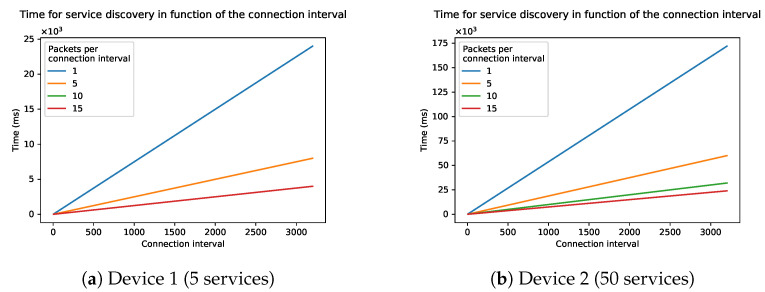
Time of the RSDP as a function of the connection interval of two devices: (**a**) Device 1 and (**b**) Device 2 with a limitation on packets per connection interval.

In a lot of commercial devices and BLE frameworks, the number of packets a device can send per connection event is limited. In the Zephyr RTOS BLE implementation, this is limited. The default setting in Zephyr is 6, but can be increased to 18. As the RSDP tries to fill each connection interval with packets, this limitation could have a great influence on the discovery speed. Figure 17 shows the influence of the connection interval on the total discovery time, taking into account this limitation of packets per connection event. In these graphs we used the physical mode 2M, as this showed the best results in previous graphs. The used MTU in these simulations is 23 which is the default value for most BLE frameworks. It is clear that the more packets sent per connection interval, the less steep the curve.

**Figure 18:**
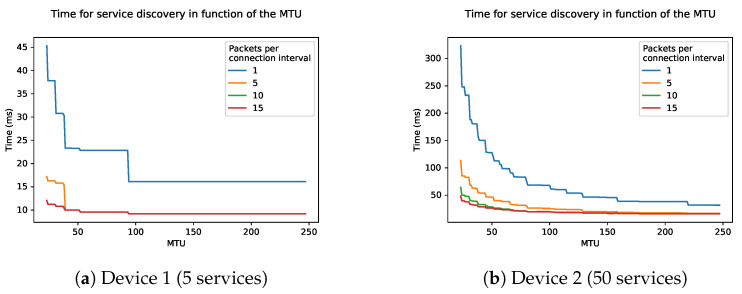
Time of the RSDP as a function of the MTU of two devices: (**a**) Device 1 and (**b**) Device 2 with a limitation on packets per connection interval.

Figure 18 shows the influence of the MTU on the total discovery time. In these graphs we used the 2M physical mode and a connection interval of 6 (7.5 ms). The curve of 10 and 15 packets per connection interval is identical. This also matches the 2M graph as seen in Figure 15. This is due to the fact that fewer than 10 packets have to be sent in order to discover everything using the RSDP on Device 1. Eventually, when the packet size is high enough, five packets will also be enough. One packet per connection interval will never be enough, as it sends a packet for all services, a packet for all characteristics, and a packet for all descriptors. Three packets per interval with a very high MTU is the minimum to send all services, characteristics, and descriptors in a single connection interval.

For Device 2, a similar phenomenon can be observed. For this complex device, it requires a lot more packets to do a full discovery. As the MTU increases the graphs also converge, except for the one where only 1 packet per connection interval is sent. In commercially available devices there is often a limit of five packets per connection interval from peripheral to central. The results of Figure 18 show that it is possible to achieve similar results as with more packets per connection interval when the MTU is higher. We can thus conclude that if there is a limitation of packets per connection interval, it is also important to keep the MTU as high as possible and the connection interval as low as possible.

### 5.3. Time Analysis

**Figure 19:**
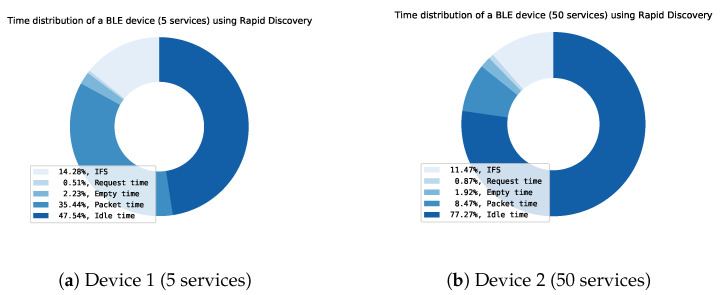
Time distribution of the RSDP of two devices: (**a**) Device 1 and (**b**) Device 2.

Figure 19 shows the time distribution of the whole service discovery with optimal parameters and no packet limit. When comparing with the original complete discovery in Figure 12, the idle time is drastically reduced. If a device has a lot of services and characteristics, the idle time will be reduced even more. The idle time is mainly caused by the remainder of the connection interval where the central sends the discovery request.

In Table 4 a comparison is made between the theoretical time needed for complete original service discovery and a complete discovery using the Rapid Service Discovery, using the optimal settings (Connection interval: 6, MTU: 247, Connection speed: 2M PHY). The speed is significantly faster, more than 1000% for Device 1 (5 services) and more than 3500% for Device 2 (50 services).

A practical implementation of this has not been made due to the complexity of doing this. We did, however, check the feasibility to implement this on a real device. When implementing this into an existing stack, access is needed to the ATT and GATT layer code of the BLE implementation, which is often a propitiatory and closed source. In Zephyr RTOS is fully open source, which means all Bluetooth layers can be modified. The file, where all ATT and GATT commands are parsed, is “Bluetooth/host/att.c” and “bluetooth/host/gatt.c”. To implement the RSDP in Zephyr an additional command with a distinct identifier should be added to the BLE central device. In turn, the peripheral has to generate all the packets to send the services, characteristics and descriptors to respond to that command. In current implementation a request from the central is followed by a response of the peripheral. This happens by registering a response callback in the BLE stack. In the RSDP however, the response consists out of multiple packets. Extra functionality has to be written in the link layer, to capture the received RSDP packets and parse these. Furthermore, this function could provide a custom callback to the request to confirm everything was discovered. To implement these complex methods, however, a lot of knowledge is required from the communication, internal structure and data management of the Zephyr framework.

## 6. Discussion

By analyzing the existing Bluetooth low energy transfer protocol and service discovery, a simulation framework was implemented. By using this framework, a theoretical analysis was made to examine the influence of different connection parameters. These tests show that the connection interval has a big influence on the discovery speed. The MTU and data length have an influence, especially when using more services and characteristics. Changing the transmission speed has little to no influence on the total time needed for the service discovery.

To validate the results of the theoretical analysis, an experimental implementation was made on physical devices. In the practical setup, we also studied the effect of using a different BLE framework to see if it has an influence on the discovery processes. The service discovery time in practice differs a small bit from the simulated one. The amount of retransmissions during the discovery process has an impact on the results. It also has shown that processing time during the discovery phase should take less time than a connection interval. The experiments, where different BLE stacks were used for central en peripheral devices, highlighted that some BLE frameworks do not provide freedom to modify all link-layer parameters. Moreover, it shows that interpretation of the BLE specification is possible leading to further optimizations, which are further explained in the next paragraph.

The simulations, practical experiments, and a thorough analysis of the Bluetooth stack, resulted in a set of best practices to optimize the service discovery process between a central and peripheral device:

- A BLE central can set the connection interval to the lowest value of 6 (7.5 ms).
- A peripheral should support as high as possible Data Length and a corresponding MTU. This leads to larger packet size and thus faster discovery. The central should set the MTU/Data Length as high as possible.
- When doing a full-service discovery, the BLE specification mentions all services should be discovered first. For each of these services handle range, characteristics should be discovered with a separate request. To optimize this, instead, all characteristics can be discovered at once by doing a read-by-type request of the whole handle range.
- The descriptor discovery not always necessary. When using BLE notifications and indications, there is a mandatory CCCD descriptor attached to the characteristic. By using this knowledge, the discovery of these specific descriptors can be skipped.
- When using different BLE stacks we can notice that the Bluetooth specification can be interpreted differently. When peripheral devices interpret the stack more loosely it can fit one byte extra. The specification says that during attribute data transactions an ATT opcode (1 byte) and ATT handle (2 bytes) are used. However, during the service discovery a data length (1 byte) field is used instead of an ATT handle leading to 1 spare byte. This can sometimes lead to fewer amounts of total packets. For example, when using the standard 23 MTU it can fit three characteristics instead of two.

The BLE service discovery can be optimized to the range of milliseconds by changing previously mentioned parameters.However, there is still room for improvement, especially in cases where more complex devices are used, and in time-critical applications. Our results have shown that the devices are mostly idle during the discovery process. Making use of these idle times leads to a significant increase in discovery speed. That is why, in this paper, we propose a Rapid Service Discovery Protocol, allowing faster and more efficient service discovery processes. By decreasing the number of requests that need to be sent, and filling connection intervals with more data, the speed of service discovery can be significantly increased.

This study did not include the discovery of “included services”, which are not often used. During the simulation, we always matched the MTU with the data length. When a larger MTU is used, fragmenting of these packets will happen. The effect of this fragmenting on the discovery process still has to be researched. Future research might also include the effect of custom services, characteristics, and descriptors on the service discovery, which were not included during this study.

## 7. Conclusions

In this study, a simulation framework was made to theoretically analyze the BLE discovery process. During this simulation, link-layer parameters were taken into account, which influence its performance, such as the MTU, connection interval, and physical mode. This simulation resulted in a set of optimal parameters to speed up the discovery process.

Validation of these results was done via an experimental analysis on physical devices. These results have shown that, when using the optimal parameters, the theoretically calculated discovery time is often influenced by packet loss/retransmissions. Additionally, we examined if there are differences between the discovery processes of different BLE stacks. These differences highlighted some important points to consider when optimizing the discovery process.

After analysis of the simulation and experimental results, a set of guidelines could be made, able to improve the speed of the discovery process.

Even after optimizing the discovery process, our results have shown the current discovery process is not efficient. For this reason, in this paper we proposed a Rapid Service Discovery Protocol, providing a faster and more efficient discovery process.

## Figures and Tables

**Table 1:** Example of a part of an attribute table.

	Handle	UUID	Permission	Attribute Value
Service Declaration	0x0010	0x2800	0x02	0x180D
Characteristic Declaration	0x0011	0x2803	0x02	Properties: read, notify Value handle: 0x0012 UUID: 0x2A37
Characteristic Value Declaration	0x0012	0x2A37	0x12	67
Descriptor Declaration	0x0013	0x2902	0x0A	0x0001
Characteristic Declaration	0x0014	0x2803	0x02	Properties: read Value handle: 0x0015 UUID: 0x2A38
Characteristic Value Declaration	0x0015	0x2A38	0x02	3

**Table 2:** Device 1 comparison practice to theory.

Conn. Interval	PHY Mode	MTU	Total Time	Theory Time	Difference
6	2M	247	0.10563	0.09778	8.03%
6	1M	247	0.11273	0.09789	15.16%
6	CODED	247	0.10587	0.09873	7.23%
6	2M	23	0.20281	0.18778	8.01%
6	1M	23	0.18851	0.18789	0.33%
6	CODED	23	0.20337	0.18873	7.76%
60	2M	247	0.97562	0.97528	0.04%
60	1M	247	1.05066	0.97539	7.72%
60	CODED	247	1.12589	0.97623	15.33%
60	2M	23	2.02463	1.87528	7.96%
60	1M	23	1.94924	1.87539	3.94%
60	CODED	23	2.0259	1.87623	7.98%

**Table 3:** Device 2 comparison practice to theory.

Conn. Interval	PHY Mode	MTU	Total Time	Theory Time	Difference
6	2M	247	0.60157	0.56278	6.89%
6	1M	247	0.57094	0.56289	1.43%
6	CODED	247	0.60838	0.56373	7.92%
6	2M	23	1.67378	1.62778	2.83%
6	1M	23	1.71068	1.62789	5.09%
6	CODED	23	1.6809	1.62873	3.20%
60	2M	247	5.92499	5.62528	5.33%
60	1M	247	5.70098	5.62539	1.34%
60	CODED	247	5.85096	5.62623	3.99%
60	2M	23	16.95142	16.27528	4.15%
60	1M	23	16.72648	16.27539	2.77%
60	CODED	23	16.87613	16.27623	3.69%

**Table 4:** Time of the Rapid Service Discovery compared with the original service discovery when using the optimal link-layer settings for both Device 1 (5 services) and Device 2 (50 services).

	Device 1 (5 Services)	Device 2 (50 Services)
Original time	97,778 μs	562,778 μs
RSDP time	9158 μs	15,758 μs
Percentage difference	1067.67%	3571.37%

## Data Availability

The data presented in this study are available on request from the corresponding author.

## References

[1] Lee J.S., Su Y.W., Shen C.C. A comparative study of wireless protocols: Bluetooth, UWB, ZigBee, and Wi-Fi. Proceedings of the IECON 2007—33rd Annual Conference of the IEEE Industrial Electronics Society.

[2] Rondón R., Gidlund M., Landernäs K. (2017). Evaluating Bluetooth Low Energy Suitability for Time-Critical Industrial IoT Applications. Int. J. Wirel. Inf. Netw..

[3] Omre A.H., Keeping S. (2010). Bluetooth low energy: Wireless connectivity for medical monitoring. J. Diabetes Sci. Technol..

[4] Zhang T., Lu J., Hu F., Hao Q. Bluetooth low energy for wearable sensor-based healthcare systems. Proceedings of the 2014 IEEE Healthcare Innovation Conference (HIC).

[5] Collotta M., Pau G. (2015). A solution based on bluetooth low energy for smart home energy management. Energies.

[6] Gomez C., Oller J., Paradells J. (2012). Overview and evaluation of bluetooth low energy: An emerging low-power wireless technology. Sensors.

[7] Tosi J., Taffoni F., Santacatterina M., Sannino R., Formica D. (2017). Performance evaluation of bluetooth low energy: A systematic review. Sensors.

[8] Kindt P., Yunge D., Diemer R., Chakraborty S. (2014). Precise Energy Modeling for the Bluetooth Low Energy Protocol. arXiv.

[9] Siekkinen M., Hiienkari M., Nurminen J.K., Nieminen J. How low energy is bluetooth low energy? Comparative measurements with ZigBee/802.15.4. Proceedings of the 2012 IEEE Wireless Communications and Networking Conference Workshops (WCNCW).

[10] Pang B.Z., Claeys T., Pissoort D., Hallez H., Boydens J. Comparative study on AFH techniques in different interference environments. Proceedings of the 2019 28th International Scientific Conference Electronics (ET).

[11] Pang B., Claeys T., Pissoort D., Hallez H., Boydens J. A Study on the Impact of the Number of Devices on Communication Interference in Bluetooth Low Energy. Proceedings of the 2020 29th International Scientific Conference Electronics (ET).

[12] Spörk M., Classen J., Boano C.A., Hollick M., Kay R. Improving the Reliability of Bluetooth Low Energy Connections. Proceedings of the International Conference on Embedded Wireless Systems and Networks (EWSN).

[13] Liu J., Chen C., Ma Y. Modeling and performance analysis of device discovery in Bluetooth Low Energy networks. Proceedings of the 2012 IEEE Global Communications Conference (GLOBECOM).

[14] Liu J., Chen C., Ma Y., Xu Y. Energy analysis of device discovery for bluetooth low energy. Proceedings of the 2013 IEEE 78th Vehicular Technology Conference (VTC Fall).

[15] Mikhaylov K. Accelerated Connection Establishment (ACE) mechanism for Bluetooth Low Energy. Proceedings of the 2014 IEEE 25th Annual International Symposium on Personal, Indoor, and Mobile Radio Communication (PIMRC).

[16] Mikhaylov K. Simulation of network-level performance for Bluetooth Low Energy. Proceedings of the 2014 IEEE 25th Annual International Symposium on Personal, Indoor, and Mobile Radio Communication (PIMRC).

[17] Miller R.B. Response Time in Man-Computer Conversational Transactions. Proceedings of the Fall Joint Computer Conference, Part I.

[18] Card S.K., Robertson G.G., Mackinlay J.D. The information visualizer, an information workspace. Proceedings of the SIGCHI Conference on Human Factors in Computing Systems.

[19] Dementyev A., Hodges S., Taylor S., Smith J. Power consumption analysis of Bluetooth Low Energy, ZigBee and ANT sensor nodes in a cyclic sleep scenario. Proceedings of the 2013 IEEE International Wireless Symposium (IWS).

[20] Lin J.R., Talty T., Tonguz O. (2015). On the potential of bluetooth low energy technology for vehicular applications. IEEE Commun. Mag..

[21] Bluetooth Special Interest Group (SIG) (2020). Bluetooth Core Specification Version 5.2. https://www.bluetooth.org/docman/handlers/downloaddoc.ashx?doc_id=478726.

[22] Bluetooth Special Interest Group (SIG) Bluetooth Core Specification Version 5.0. https://www.bluetooth.org/DocMan/handlers/DownloadDoc.ashx?doc_id=421043.

[23] Bluetooth Special Interest Group (SIG) (2014). Bluetooth Core Specification Version 4.2. https://www.bluetooth.org/DocMan/handlers/DownloadDoc.ashx?doc_id=286439.

[24] Nordic Semiconductors nRF Sniffer for Bluetooth LE. https://www.nordicsemi.com/Software-and-tools/Development-Tools/nRF-Sniffer-for-Bluetooth-LE.

[25] Nordic Semiconductors nRF52840. https://www.nordicsemi.com/Products/Low-power-short-range-wireless/nRF52840.

[26] Texas Instruments LP-CC2652RB. https://www.ti.com/tool/LP-CC2652RB.

[27] NCC Group Sniffle. https://github.com/nccgroup/Sniffle.

[28] KimiNewt PyShark. https://github.com/KimiNewt/pyshark.

[29] Google BluetoothGatt. https://developer.android.com/reference/android/bluetooth/BluetoothGatt.

[30] Google Config Android Source Code. https://android.googlesource.com/platform/packages/apps/Bluetooth/+/refs/heads/master/res/values/config.xml.

